# A Case of Autoimmune Hepatitis Initially Manifesting as Hepatic Encephalopathy

**DOI:** 10.7759/cureus.62890

**Published:** 2024-06-22

**Authors:** Shengmin Yang, Ning Zhang, Xiang Li, Yunlong Li, Liangrui Zhou, Yuchen Wei, Kanghao Zhou, Hui Pan, Lin Kang

**Affiliations:** 1 Key Laboratory of Endocrinology of National Health Commission, Department of Endocrinology, State Key Laboratory of Complex Severe and Rare Diseases, Peking Union Medical College Hospital, Peking Union Medical College, Chinese Academy of Medical Sciences, Beijing, CHN; 2 Department of Geriatrics, Peking Union Medical College Hospital, Peking Union Medical College, Chinese Academy of Medical Sciences, Beijing, CHN; 3 Department of Internal Medicine, Peking Union Medical College Hospital, Peking Union Medical College, Chinese Academy of Medical Sciences, Beijing, CHN; 4 Department of Pathology, Peking Union Medical College Hospital, Peking Union Medical College, Chinese Academy of Medical Sciences, Beijing, CHN

**Keywords:** case report, multidisciplinary approach, collateral circulation, hepatic encephalopathy, elderly, autoimmune hepatitis

## Abstract

Autoimmune hepatitis (AIH) is a T-cell-mediated liver disease characterized by elevated transaminases, circulating autoantibodies, hypergammaglobulinemia, and interface hepatitis. A 66-year-old female patient visited our department due to recurrent episodes of altered consciousness, sleep-wake inversion, and asterixis, indicating hepatic encephalopathy (HE). Her liver biopsy results clearly demonstrated interface hepatitis. The patient's severe HE does not parallel her relatively stable liver function and was attributed to a wide retroperitoneal collateral vein shunting blood directly into the inferior vena cava, bypassing the liver, and allowing excess neurotoxins to enter the central nervous system. Due to the unfavorable benefit-risk ratio of embolization and the patient's stable liver function, non-invasive treatments were adopted, and prednisolone was discontinued. The patient experienced no further episodes of HE thereafter. To the best of our knowledge, this is the first AIH case with a spontaneous portosystemic shunt directly shunting blood into the inferior vena cava. A crucial lesson from this case is that when HE cannot be fully explained by liver dysfunction, abdominal CT scans should be carefully inspected for possible anatomical variations. This case also underscores the importance of a multidisciplinary approach in managing AIH in elderly patients, who may benefit more from a tailored treatment regimen rather than strictly following standard treatment guidelines.

## Introduction

Autoimmune hepatitis (AIH) is a progressive liver disorder characterized by T-cell-mediated destruction of hepatocytes, predominantly affecting women [1]. The worldwide aggregated prevalence of AIH is reported to be 15.65 cases per 100,000 individuals (95% confidence interval (CI), 13.42-18.24) [2], being lower in Asians as compared to Caucasians [3]. Clinically, AIH may manifest asymptomatically or with non-specific symptoms such as fatigue, abdominal discomfort, or jaundice, complicating its early diagnosis. Regarding laboratory examinations, AIH typically presents with elevated transaminases, circulating autoantibodies, and hypergammaglobulinemia [4]. Serologically, while alanine aminotransferase (ALT) and aspartate aminotransferase (AST) are significantly elevated, alkaline phosphatase (ALP) and γ-glutamyl transferase (GGT) levels may remain normal or only mildly elevated in most cases. During severe episodes, total bilirubin levels may significantly increase. Immunoglobulin G (IgG) levels are generally elevated, while IgM and IgA levels remain normal. Regarding the autoantibody profile, antinuclear antibody (ANA) and anti-smooth muscle antibody (SMA) are typically present for type 1 AIH, which accounts for about 90% of AIH cases. Type 2 and type 3 AIH are less common. Type 2 is characterized by anti-liver kidney microsome type 1 (anti-LKM-1) and anti-liver cytosol type 1 (anti-LC1) antibodies, while type 3 is characterized by antibodies against soluble liver antigen (SLA) [5]. AIH is typically negative for anti-mitochondria antibody (AMA), a marker for primary biliary cholangitis.

A confirmed diagnosis of AIH requires histopathological evidence [6]. Specifically, interface hepatitis, a hallmark of AIH, involves inflammatory infiltration and necrosis extending from the portal areas into the surrounding hepatic parenchyma [7]. This feature is diagnostic of AIH, especially when accompanied by bridging necrosis, rosette formation of hepatocytes, and emperipolesis [7,8]. Approximately one-third of patients already present with cirrhosis at diagnosis [9], and around 60% eventually develop cirrhosis [10]. Liver cirrhosis is characterized by progressive portal hypertension, resulting in the spontaneous formation of portosystemic shunts. These shunts act as "release valves" to reduce portal pressure by diverting venous blood away from the liver [11]. As these shunts enlarge, complications such as hepatic encephalopathy (HE), variceal bleeding, portal vein thrombosis, and liver function deterioration emerge, constituting the so-called portosystemic shunt syndrome [12]. Here, we report a case of a patient with AIH who initially manifested with HE and presented as typical interface hepatitis on biopsy. The patient's HE symptoms are more severe than expected given her liver function. This is attributed to her narrow portal vein, which has high pressure promoting collateral circulation formation. In turn, the presence of a wide collateral vein diverts blood away from the liver, directly entering the inferior vena cava. This results in an excess of neuroactive substances entering the central nervous system.

## Case presentation

A 66-year-old female visited the Department of Geriatrics at Peking Union Medical College Hospital (PUMCH) for recurrent episodes of altered consciousness over the past month. She also suffered from sleep-wake inversion, deteriorating mental health (including irritability and anxiety), and pruritus over the past six months. Following a quarrel with others and a delayed meal, the patient exhibited symptoms of somnolence, profuse sweating, urinary incontinence, flaccid extremities, and bilateral hand tremors in the evening. An emergency assessment yielded the following arterial blood gas analysis: pH 7.48, PaCO2 34 mmHg, PaO2 114 mmHg, bicarbonate 26.6 mmol/L, and normal electrolytes. Laboratory findings included normal blood cell counts, elevated ALT at 69 U/L (reference range: 7-40 U/L), AST at 173 U/L (reference range: 13-35 U/L), ALP at 191 U/L (reference range: 50-135 U/L), total bilirubin at 41.6 µmol/L (reference range: 5.1-22.2 µmol/L), conjugated bilirubin at 18 µmol/L (reference range: <6.8 µmol/L), blood ammonia at 47.9 µmol/L (reference range: 11-32 µmol/L), and decreased albumin at 28 g/L (reference range: 35-52 g/L). This patient had roughly normal coagulation function, with prothrombin time at 13.2 seconds (reference range: 10.4-12.6 seconds), activated partial thromboplastin time at 26.3 seconds (reference range: 23.3-32.5 seconds), and fibrinogen at 1.84 g/L (reference range: 1.80-3.50 g/L). The patient presented with ANA 1:100 (homogeneous pattern), while other autoimmune antibodies such as SMA, anti-glycoprotein 210 (gp210), anti-nuclear auto-antigen Sp-100, anti-Smith, anti-dsDNA, anti-SSA, anti-SSB, anti-RNP, and anti-Jo-1 were all negative. Abdominal ultrasound showed diffuse changes in liver parenchyma. An abdominal CT scan documented thin portal veins and newly formed collateral circulation. Treatment with arginine and ornithine aspartate ameliorated the neurological issues within one day, preventing recurrence thereafter. Prednisolone was initiated at 30 mg daily, but significant side effects ensued (including Cushing's appearance, aggravated edema, and steroid-induced diabetes), necessitating a gradual reduction to a maintenance dose of 5 mg daily. Her past medical history includes hypertension, Hashimoto's thyroiditis, cholecystectomy, and right knee arthroplasty. Her personal history reveals occasional alcohol consumption (400 g alcohol per week) and smoking, which ceased 20 years ago.

At admission, physical examination revealed an obese figure (BMI 30.5 kg/m²), Cushing's appearance, episodes of asterixis, palmar erythema, and multiple spider angiomas on her chest and back. Her abdomen was distended without tenderness or rebound tenderness; the liver and spleen were not palpable below the rib cage. Edema was noted in her eyelids, with symmetrical pitting edema below the knees. Laboratory examination showed normal blood counts, transaminases (ALT 17 U/L, AST 31 U/L), GGT (16 U/L, reference range: 5-45 U/L), and ALP (102 U/L), but elevated blood ammonia (68-110 μmol/L, reference range: 11-32 μmol/L), bilirubin (total bilirubin 36.0 μmol/L, direct bilirubin 17.3 μmol/L), total bile acid (67.3 μmol/L, reference range: <10 μmol/L), and liver fibrosis markers (hyaluronic acid 515 ng/mL, reference range: <120; procollagen type III N-terminal propeptide 30 ng/mL, reference range: <15). In addition, decreased cholinesterase (2.3 kU/L, reference range: 7-45 kU/L), creatinine (44 μmol/L, reference range: 45-83 μmol/L), albumin (32 g/L), and prealbumin (74 mg/L, reference range: 200-400 mg/L) were observed. The patient had a normal erythrocyte sedimentation rate of 21 mm/h and a normal C-reactive protein beneath 0.5 mg/L. Fecal occult blood testing was negative for three times. Screening for hepatotropic viruses revealed negative HBV-DNA, HCV-RNA, CMV-DNA, and EBV-DNA. The patient had a positive ANA of 1:80 (homogeneous speckled pattern), while all other autoimmune antibodies were negative. A decrease in C4 (0.068 g/L, reference range: 0.1-0.4 g/L) and a mild increase in IgA (4.42 g/L) were detected, while C3 (0.779 g/L, reference range: 0.73-1.46 g/L) and IgG (16.62 g/L, reference range: 7-17 g/L) were within normal limits. Serum protein electrophoresis indicated decreased albumin (49.2%) and increased gamma globulins (26.2%). Endocrine and metabolic assessments confirmed normal levels of folate, vitamin B12, thyroid function, hemoglobin A1c (5.0%), and ceruloplasmin. Total 25-hydroxy vitamin D (21.2 ng/mL, reference range: >30 ng/mL) was insufficient.

**Figure 1:**
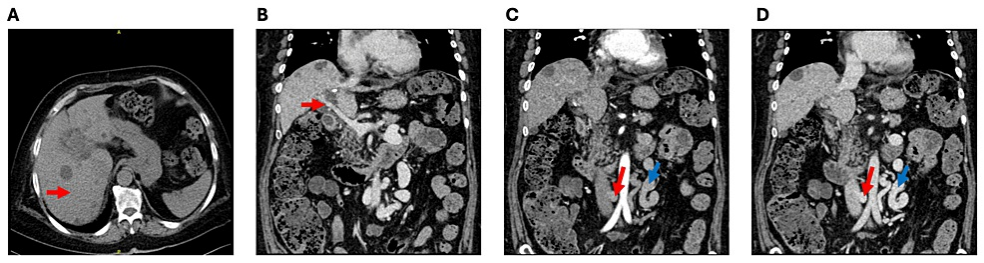
Contrast-enhanced abdominal CT (A) Liver (axial view, non-contrast): the red arrow points to the cirrhotic liver. (B) Portal vein (coronal view, portal phase): the red arrow points to the narrow portal vein. (C) Collateral circulation (coronal view, arterial phase): the red arrow points to the wide collateral vein, and the blue arrow points to other parts of the collateral vein. (D) Collateral circulation (coronal view, portal phase): the red arrow points to the wide collateral vein, and the blue arrow points to other parts of the collateral vein.

**Figure 2:**
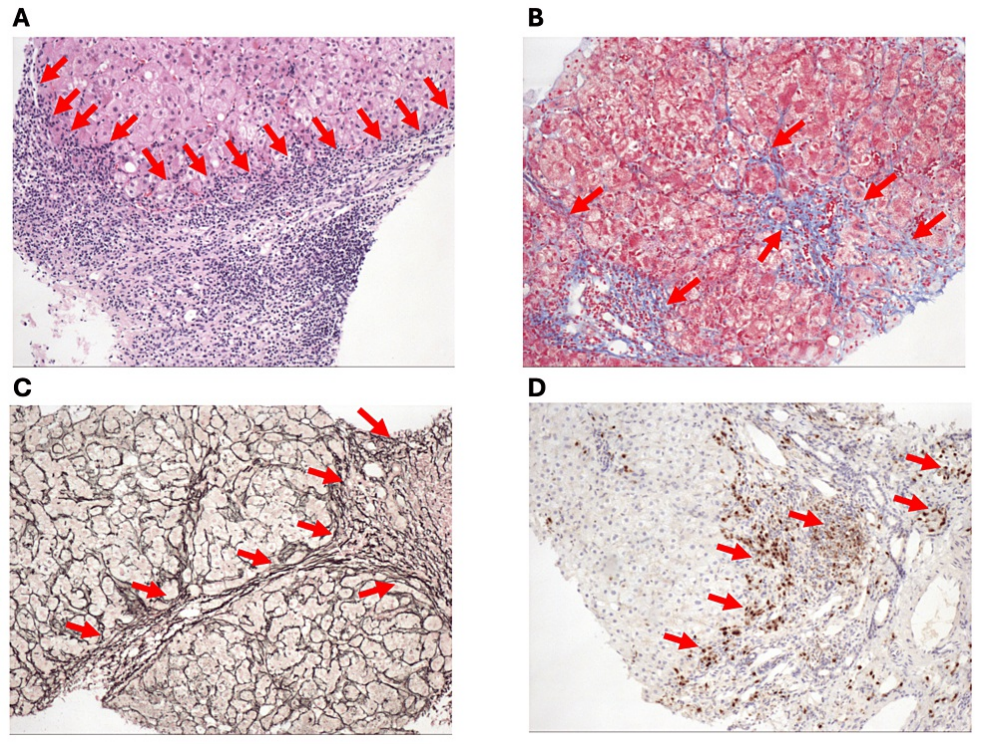
Liver biopsy results (200× enlarged) (A) Hematoxylin and eosin stain: red arrows show interface hepatitis. (B) Masson trichrome stain: red arrows show increased fibrosis with cord-like bridges. (C) Silver stain: red arrow shows increased fibrosis. (D) Multiple myeloma oncogene 1 (MUM1) stain: red arrow shows MUM1-expressing plasma cells/plasma cell precursors.

An enhanced abdominal CT (Figure 1) with portal vein 3D reconstruction showed a reduced liver volume with less smooth contours and widened fissures (Figure 1A). Dilated intrahepatic bile ducts at the hepatic hilum and multiple cysts within the liver were also observed. The portal vein's main trunk (0.7 cm) and branches were thin (Figure 1B), surrounded by multiple tortuous vascular shadows, suggesting a possible spongy transformation. A wide retroperitoneal spontaneous portosystemic shunt (SPSS) was identified, measuring 1.75 cm in diameter (Figure 1C, 1D). Liver elastography recorded a liver parenchymal elasticity value of 10.3 kPa. Deep vein ultrasound of the lower limbs showed no significant thrombosis. An echocardiogram reported a left ventricular ejection fraction of 69%, an enlarged left atrium (anteroposterior diameter 4.1 cm), and a widened ascending aorta (3.5 cm) with mild aortic valve stenosis and regurgitation. Head MR angiography (MRA) revealed no significant abnormalities. Liver biopsy (Figure 2) showed portal tracts with enlargement, fibrous tissue proliferation accompanied by moderate lymphoplasmacytic infiltration, and bile duct proliferation. Significant interface hepatitis was observed, with some areas showing cord-like bridging fibrous tissue dividing liver lobules, multiple small focal areas of hepatocellular necrosis, and individual hepatocytes with macrovesicular steatosis, suggestive of chronic hepatitis (G2-3/S3), thereby supporting the diagnosis of AIH.

A multidisciplinary discussion involving geriatrics, gastroenterology, general surgery, immunology, endocrinology, nutrition, and pharmacy considered the following: The patient's elevated transaminases, positive ANA, and distinctive interface hepatitis well supported the diagnosis of AIH. A personal history of alcohol consumption may have also aggravated the patient's baseline liver status. The patient's narrow portal vein and the ensuing formation of retroperitoneal collateral circulation lead to blood flow bypassing the liver and directly entering the inferior vena cava, explaining her susceptibility to HE. Although angiographic embolization of collateral vessels could reduce portosystemic shunting and potentially improve HE, there is a risk of developing esophageal and gastric varices which may result in lethal gastrointestinal bleeding. Hence, given the low benefit-risk ratio of angiographic embolization and the patient's relatively stable liver function, the primary treatment focus should be taking non-invasive measures to prevent the recurrence of HE. Given that glucocorticoid therapy does not reverse the pathological changes associated with cirrhosis and has significant side effects and normalized markers for systematic inflammation (such as ESR and CRP), it is advisable to discontinue prednisolone. Medication for HE prevention (ornithine aspartate granules one packet twice daily, oral lactulose 10 g twice daily, and intermittent glycerin enemas), liver protection (glutathione 50 mg three times daily), diuresis (furosemide 20 mg daily, spironolactone 20 mg twice daily), and bile stasis reduction (ursodeoxycholic acid 250 mg twice daily) were administered. These treatments led to daily defecation, significantly reduced edema, improved fitness status, no further episodes of consciousness disturbances, and reduced bilirubin levels (total bilirubin 23.6 μmol/L, conjugated bilirubin 13.1 μmol/L). For steroid-induced diabetes, daily subcutaneous insulin glargine injection at 6U kept fasting blood glucose between 6 mmol/L and 8 mmol/L and postprandial blood glucose between 8 mmol/L and 14 mmol/L.

## Discussion

AIH was first reported in young women, but since the late 1990s, an increasing number of cases (approximately 20-25%) have been identified in individuals aged 60 and older [13]. Consequently, AIH should be considered in elderly patients with acute or chronic liver disease. Moreover, those diagnosed after the age of 60 are more likely to present with cirrhosis, suggesting a high risk of decompensation [13]. The majority of patients have an insidious onset, presenting only with non-specific symptoms such as fatigue. However, a minority of AIH cases have an acute presentation, some of which are indeed exacerbations of chronic AIH that progress to acute liver failure. AIH is predominantly characterized by hepatocellular injury, evidenced by elevated serum levels of ALT and AST, while serum ALP and GGT levels, which are significantly elevated in primary biliary cholangitis, are roughly normal or mildly elevated in AIH. During severe or acute episodes, total bilirubin levels can be significantly elevated. Elevated IgG levels are also typical in AIH, but lower serum IgG levels at diagnosis may correlate with a favorable prognosis [14]. Pathologically, AIH is typified by interface hepatitis, characterized histologically by inflammation extending from the portal tracts/fibrous septa to the lobules, leading to piecemeal necrosis and the dropout of adjacent hepatocytes [15]. Untreated or inadequately managed AIH can progress to cirrhosis and end-stage liver disease, underscoring the importance of timely treatment.

HE is a condition characterized by changes in consciousness, behavior, cognition, and psychomotor function, resulting from the accumulation of neurotoxins (such as ammonia, manganese, and short-chain fatty acids) due to hepatocellular dysfunction (synthetic HE) or portosystemic shunting (type B HE or bypass HE). Our patient, a 66-year-old female, exhibited clinical manifestations of typical HE and classic presentations of AIH (including elevated ALT and AST levels, positive ANA, and histological features of interface hepatitis). The patient's severe HE, disproportionate to her liver function tests, was attributed to her unique anatomical variations, specifically the extensive collateral circulation. This anatomical anomaly closely mimics those who have undergone transjugular intrahepatic portosystemic shunt (TIPS) implantation, facilitating the shunting of ammonia-rich blood from the intestines directly into the systemic circulation, bypassing hepatic detoxification, and leading to neurotoxicity. The portal vein narrowing in this patient may be explained by the presence of extensive fibrosis in cirrhosis, despite most patients with cirrhosis presenting with an enlarged portal vein [16]. Extensive retroperitoneal collateral circulation and an SPSS developed in this patient to compensate for the increased portal pressure in the narrowed portal vein. A literature review of "spontaneous portosystemic shunt" and "autoimmune hepatitis" in MEDLINE and the Cochrane Library revealed that AIH with SPSS formation is uncommon [17,18]. Most reported cases of SPSS involved patients with virus-related hepatic cirrhosis [19,20] or cirrhosis secondary to nonalcoholic steatohepatitis [21]. To the best of our knowledge, this is the first reported case of SPSS formation in AIH with extensive retroperitoneal collateral circulation. The other two cases of AIH-related SPSS involved a recanalized umbilical vein [17] and an intrahepatic SPSS [18], respectively. Moreover, the prevalence of SPSS increases over time in patients with cirrhosis, and hepatic function gradually worsens upon SPSS formation [22,23]. Specifically, Saad classified portosystemic shunt syndrome into three stages according to its natural disease course [12]. In the early stage (A), patients are asymptomatic with well-preserved hepatic function and large SPSS. In the late stage (B), patients experience symptoms, including recurrent or persistent HE, while maintaining fairly preserved liver function. The terminal or end stage (C) is marked by hepatic failure, HE, ascites, and jaundice, with thrombocytopenia commonly observed in these patients. Therefore, this patient has progressed to stage B and is showing early signs of stage C, like ascites.

In China, hepatitis B virus infection is the leading cause of cirrhosis in the elderly, followed by alcoholic cirrhosis. Cirrhosis due to AIH is rare among elderly individuals in China. The patient primarily presented with HE, and abdominal imaging confirmed the presence of a significant SPSS, which was identified as the main cause of encephalopathy. Additionally, the patient was frail and had multiple chronic conditions. Guidelines for single diseases have limited applicability in elderly patients with frailty and multiple comorbidities. Current clinical guidelines and studies often do not encompass the elderly or those with complex chronic conditions. The standard treatment of SPSS includes interventional radiology procedures like balloon-occluded retrograde transvenous obliteration (BRTO), which effectively treats refractory HE with low rebleeding rates. New techniques such as plug-assisted retrograde transvenous obliteration (PARTO) and coil-assisted retrograde transvenous obliteration (CARTO) have also proved effective in clinical practice. Although experts recommend closing large SPSS when recurrent HE is observed [12,24], this patient is unlikely to tolerate perioperative complications and the drastic increase in portal vein pressure following abrupt shunt closure. Although the standard treatment for AIH is a combination of glucocorticoids and azathioprine [4], prednisolone was discontinued due to its inability to curb cirrhosis and its severe side effects. In addition, this patient was unlikely to tolerate the side effects of azathioprine. Therefore, treatment decisions for this patient were based on functional status, personal preferences, and a comprehensive assessment of treatment risks and benefits and were made through shared decision-making between the doctor and patient. Preserving functional status and quality of life in elderly patients is a key goal in geriatric medicine. Therefore, non-invasive measures to prevent HE were adopted. In particular, medication for lowering blood ammonia levels, protecting the liver, promoting diuresis, and reducing bile stasis proved effective. The patient's condition stabilized with significant improvements in bilirubin levels, reduced edema, and no further episodes of altered consciousness. Managing elderly patients with rare diseases such as AIH and complex comorbidities requires individualized treatment plans based on comprehensive geriatric assessments. As the population ages, the number of elderly patients is expected to rise, necessitating a personalized approach to treatment. By sharing this case, we aim to gather clinical experience, learn, and strive for the most optimal treatment strategies for elderly patients.

## Conclusions

We present an intriguing AIH case in which symptoms of HE do not parallel the extent of liver function deterioration due to unique anatomical variations. This case highlights the need for personalized, multidisciplinary care in managing AIH with HE, particularly in frail elderly patients.
